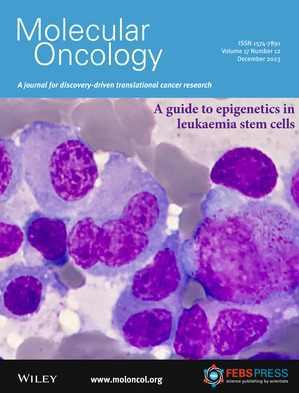# Issue Information

**DOI:** 10.1002/1878-0261.13242

**Published:** 2023-12-07

## Abstract

The current issue features a ‘Guide to’ article on epigenetics in leukemia stem cells and several research articles on hematologic malignancies. The cover image shows May‐Grünwald‐Giemsa staining of a multiple myeloma patient‐derived bone marrow aspirate, revealing atypical plasmatic cells with the presence of anisocytosis and multinuclearity. Read the full articles from the groups of Brian J.P Huntley in pp. 2493–2506 and Isabel Marzo pp. 2507–2525.

Image credit: Dr. R.Díez.